# Protective effects of engineered *Lactobacillus johnsonii* expressing bovine granulocyte-macrophage colony-stimulating factor on bovine postpartum endometritis

**DOI:** 10.3389/fvets.2024.1418091

**Published:** 2024-08-08

**Authors:** Jing Guo, Xu Cao, Zhiqiang Li, Caiyu Wang, Chengkun Zhong, Simin Wang, Zhile Fan, Jing Zhao, Jun Wang, Yi Fang, Hongyu Liu, He Ding, Xin Ma, Wenfa Lu

**Affiliations:** ^1^Key Lab of the Animal Production, Product Quality and Security, Ministry of Education, Jilin Agricultural University, Changchun, China; ^2^Jilin Provincial International Joint Research Center of Animal Breeding and Reproduction Technology, Jilin Agricultural University, Changchun, China; ^3^Jilin Province Key Laboratory of Beef Cattle Germplasm Resources Utilization, Jilin Agricultural University, Changchun, China

**Keywords:** postpartum endometritis, GM-CSF, *Lactobacillus johnsonii*, protective effects, inflammation

## Abstract

**Introduction:**

Postpartum endometritis is a prevalent reproductive disorder in bovines, leading to a prolonged open period, infertility, and other complications. While *Lactobacillus* strains can mitigate these conditions by reducing uterine inflammation, their effectiveness is limited due to a lack of direct anti microbial action and extended treatment duration. This study aimed to construct a recombinant *Lactobacillus johnsonii* strain expressing bovine Granulocyte-macrophage colony-stimulating factor (GM-CSF) to evaluate its potential in reducing postpartum uterine inflammation.

**Methods:**

The recombinant *Lactobacillus johnsonii* strain was engineered to express bovine GM-CSF and administered to pregnant mice via vaginal perfusion. Postpartum endometritis was induced using *E. coli* infection, and the protective effects of the engineered strain were assessed. Inflammatory markers (IL-6, IL-1β, TNF-α), myeloperoxidase (MPO) activity, and nitric oxide (NO) concentration were measured. Histological examination was performed to evaluate uterine morphology and pathological damage.

**Results:**

The recombinant *L. johnsonii* strain expressing GM-CSF significantly reduced inflammation levels induced by *E. coli* infection in the uterus. This reduction was evidenced by decreased expression of IL-6, IL-1β, TNF-α, as well as reduced MPO activity and NO concentration. Histological examination revealed improved uterine morphology and reduced pathological damage in mice treated with the recombinant GM-CSF strain. Crucially, the recombinant strain also exerts beneficial effects on bovine endometritis by reducing levels of inflammatory cytokines, suggesting a beneficial effect on clinical bovine endometritis.

**Conclusion:**

The recombinant *Lactobacillus johnsonii* expressing GM-CSF demonstrated protective effects against postpartum endometritis in bovines by reducing inflammatory cytokines. The findings indicate the potential clinical application of this engineered strain in preventing postpartum uterine inflammation, offering a novel and effective protective option for related disorders and improving bovine reproductive efficiency.

## 1 Introduction

Endometritis is one of the main diseases of bovine reproductive disorders, which can damage the reproductive ability of cows and accelerate their elimination. At the same time, it can bring huge economic losses to the livestock industry (1). Typically, 20 to 40% of cattle develop acute clinical uterine disease within a week from parturition (metritis), while 20% have a persistent clinical disease 3 weeks after calving (endometritis). Additionally, approximately 30% suffer from a chronic subclinical inflammation of the uterus (subclinical endometritis) (2). Currently, antibiotic therapy represents the prevailing approach for treating endometritis. However, the side effects of long-term antibiotic treatment, such as antibiotic resistance and antibiotic residue, have yielded unsatisfactory treatment effects. Consequently, addressing the side effects of antibiotics has prompted an increased emphasis on endometritis prevention. The most common preventive measures include enhancing dietary management or incorporating vitamins and minerals into the diet (3, 4). Unfortunately, the efficacy of these methods remains limited. Hence, there is an urgent need to identify an effective protective strategy against endometritis that can reduce its incidence rate and circumvent the adverse effects of antibiotics.

Neutrophil function is vital for the innate immunity in the reproductive tract of dairy cows. Following parturition, there is an immediate decline in neutrophil counts within the circulatory system, likely attributed to the migration of these cells towards sites of inflammation, including the postpartum uterus and mammary gland (5). Additionally, a transient state of immunosuppression occurs postpartum, characterized by impaired neutrophil phagocytosis and attenuated oxidative burst activity (6, 7). This reduction in both neutrophil abundance and functional capacity is considered a primary factor contributing to the heightened risk of retained placenta, metritis, subclinical endometritis, and mastitis (8–10). Granulocyte-macrophage colony-stimulating factor (GM-CSF) can be produced by various cell types, such as macrophages, fibroblasts, activated T-lymphocytes, natural killer cells, mast cells and so on (11). It serves as a potent hematopoietic growth factor, stimulating the expansion and maturation of monocyte-macrophages, dendritic cells, and granulocytes derived from hematopoietic progenitor cells (12). GM-CSF is widely distributed within the female reproductive tract and regulates a multitude of myeloid leukocytes within the uterus and decidual tissue. Moreover, it acts as a local mediator involved in the migration and activation of endometrial macrophages, granulocytes, and dendritic cells (13). GM-CSF also plays a role in tissue remodeling during acute structural changes associated with embryo implantation, placental development, and postpartum endometrial regeneration (14). However, the expensive price of GM-CSF has limited its application in veterinary clinical practice.

As a substitute for antibiotics, probiotics offer a broad spectrum of applications in the treatment of animal diseases resulting from bacterial infections. Lactic acid bacteria (LAB) represent the predominant microbial population within the healthy reproductive tract of cows (15). Notably, *Lactobacillus johnsonii*, isolated from the bovine vagina, has demonstrated specific adherence to the epithelium and the production of inhibitory substances (16). Our prior investigations have revealed that the *Lactobacillus johnsonii* strain obtained from bovine uterine secretions exhibits remarkable capabilities in preventing endometritis and exerts potent anti-inflammatory effects (17). However, using probiotics as a substitute for antibiotics may suffer from unstable effectiveness, limited applicability, lack of direct antimicrobial action, and longer treatment durations. Recombinant lactic acid bacteria have been successful in expressing numerous bacterial and viral antigens, thereby contributing to the enhancement of microbial homeostasis within the uterine environment (18, 19). Therefore, in the present study, we constructed a recombinant *Lactobacillus johnsonii* expressing bovine GM-CSF. Subsequently, this strain was administered to pregnant mice via vaginal perfusion, then induced postpartum endometritis mouse model, finally evaluated the effects of recombinant GM-CSF *Lactobacillus johnsonii* on the protective effects of postpartum endometritis mouse model. And also the GM-CSF strain demonstrated a favorable therapeutic efficacy against postpartum endometritis in bovines.

## 2 Materials and methods

### 2.1 Animal

A total of 122 healthy BALB/c mice (8 weeks old, weighing 25–30 g) was employed in the present study. All animal experimental procedures were performed in accordance with the Regulations for Animal Experimentation of Jilin Agriculture University (JLAU08201409), and the animal facility was based on the National Institutes of Health Guide for the Care and Use of Laboratory Animals (NIH Publications No. 8023).

### 2.2 Bacterium and plasmid

*Lactobacillus johnsonii* (stored in the China General Microbiological Culture Collection Center: GU428184.1) isolated from the uterine secretion of healthy dairy cows by our laboratory. The *Lactobacillus johnsonii* strain was incubated anaerobically at 37°C for 8 h to recover. Then 100 μL of the bacterial suspension was inoculated onto an MRS agar plate and cultured anaerobically at 37°C for 20 h. A single colony was picked and inoculated into 400 mL of MRS broth, which was then cultured to an OD of 0.5. The culture was chilled in an ice bath for 25 min and centrifuged at 4°C at 7,000 rpm for 8 min, resuspended in 20 mL of EPWB, followed by centrifugation at 4°C at 7,000 rpm for 8 min. This process was repeated twice. The pellet was resuspended in 15 mL of EPB, centrifuged again at 4°C at 7,000 rpm for 8 min, and the supernatant was discarded once more. The pellet was then resuspended in 2 mL of EPB, aliquoted into 200 μL per tube, and stored at −80°C for future use.

The *Escherichia coli-Lactobacillus* shuttle vector pPG612, is a type of cell-surface expression plasmid that contains an anchoring matrix-encoding pgsA gene, and it was derived from *Bacillus subtilis* behind the target gene. The pPG includes the ssUSP secretion signal before the target gene to ensure target protein secretion.

### 2.3 Construction of recombinant *Lactobacillus johnsonii* expressing the GM-CSF

**Figure 1:**
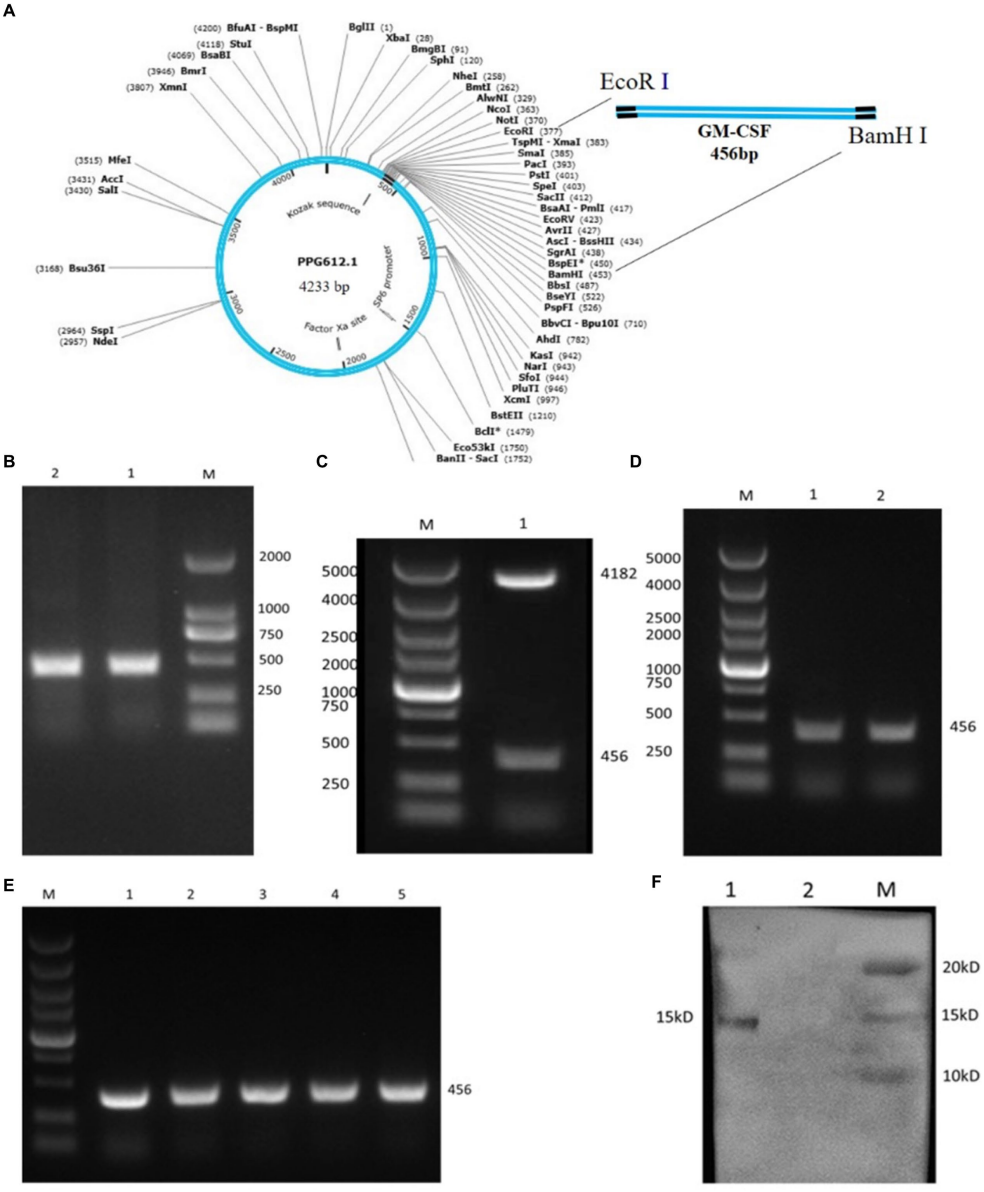
Construction of the *L. johnsonii* expressing bovine GM-CSF (Lc-pPG-GM-CSF). **(A)** Schematic diagram of the recombinant plasmid pPG612-GM-CSF. **(B)** GM-CSF gene amplification products, M: DL2000 marker; 1, 2: GM-CSF gene amplification products. **(C)** Restriction enzyme assay of recombinant plasmid pPG612-GM-CSF. M: DL5000 marker; 1: pPG-GM-CSF restriction enzyme digestion product. **(D)** PCR test results of recombinant *Lactobacillus*, M: DL5000 marker; 1, 2: Lc-pPG-GM-CSF plasmid PCR product. **(E)** Genetic stability test results of recombinant *Lactobacillus*, M: DL5000 marker, 1–5: PCR amplification products of recombinant bacteria 1st, 10th, 20th, 30th and 40th generations. **(F)** Detection of protein expression of recombinant *Lactobacillus*; M: protein marker; 1: Lc-pPG-GM-CSF recombinant *lactobacillus* protein extract; 2: *Lactobacillus johnsonii* protein extract control.

The bovine GM-CSF gene (NM_174027.2) was artificially synthesized and inserted into the vector PUC57 (Shanghai Sangon Biological Engineering and Technology Service Co., Ltd.). The length of the GM-CSF gene is 456 bp and includes GM-CSF gene coding sequence (sequences bp 10–441), two stop codon (TAATAG), restriction site EcoR I (gaattc), EcoR V (ggatcc), and a pair of protective bases (GCC and CGC) (Figure 1C).

To generate recombinant *Lactobacillus johnsonii* expressing the GM-CSF (L-GM-CSF), the DNA sequence for bovine GM-CSF was inserted into pPG-612 by restriction site EcoR I and EcoR V, followed by the formation of pPG-612-GM-CSF recombinant plasmids. The constitutive expression plasmid pPG-612-GM-CSF was transformed into *Lactobacillus johnsonii* competent cells by electroporation (Electroporator 2510, Eppendorf) under 2.1 kV 3 ms. The *Lactobacillus johnsonii* competent cells were thawed in an ice-water slurry for 10 min. Then, 5 μL of the pPG-GM-CSF recombinant plasmid was mixed gently with 100 μL of the *Lactobacillus johnsonii* competent cells. After incubating on ice for 5 min, transferred to a pre-chilled electroporation cuvette and allowed to rest for 2 min before applying a single electric pulse of 2.1 kV for 3 ms. Immediately after electroporation, the cells were chilled on ice for 10 min. The transformed cells were then transferred into 1,000 mL of MRS broth containing 15% sucrose and incubated anaerobically at 37°C for 2 h. The culture was centrifuged at 3,000 rpm 10 min, resuspended in 200 μL of MRS broth and spread onto an MRS agar plate containing 10 μg/mL of chloramphenicol. The plate was incubated anaerobically at 37°C overnight. Then, the treated *Lactobacillus johnsonii* was cultured in MRS broth medium contain 0.3 M sucrose, and the recombinant strain Lc-pPG-GM-CSF was obtained on MRS agar medium containing 10 μg/mL chloromycetin. *Lactobacillus johnsonii* containing wild-type pPG612 (Lc-pPG) was used as a vector control, and non-transformed wild-type *Lactobacillus johnsonii* was used as a negative control in all subsequent assays.

### 2.4 Western blot analysis

Expression of the recombinant plasmid Lc-pPG-GM-CSF in *Lactobacillus johnsonii* was detected using western blotting analysis. Briefly, Lc-pPG-GM-CSF and Lc-pPG were grown in basal MRS medium supplemented with 10 μg/mL chloramphenicol. Xylose was added to the culture medium to a final concentration of 10 g/L to induce GM-CSF expression. After induction at 37°C for 20 h, approximately 1 × 10^8^ cell pellets were examined using sodium dodecyl sulphate-polyacrylamide gel electrophoresis (SDS-PAGE) and transferred to a nitrocellulose membrane. The membrane was blocked with 3% BSA and incubated with bovine GM-CSF antibody (Bioss, Beijing, China) diluted 1:1,000 with phosphate-buffered saline (PBS; Bioss, Beijing, China) overnight at 4°C. Affinity-purified horseradish peroxidase (HRP)-conjugated mouse anti-bovine immunoglobulin G (IgG; Bioss, Beijing, China) was used as the secondary antibody. The blot was visualized using chemiluminescence detection with a western ECL substrate (Thermo Scientific) using an Amersham Imager 600 (GE Healthcare, Boston, United Kingdom).

### 2.5 Animal models and treatments

One hundred and twenty-two female BALB/c mice (8 weeks old, weighing 25–30 g) were sourced from Liaoning Changsheng Biotechnology Co., Ltd. The mice were housed in specific pathogen-free enclosures within the laboratory animal facility at Jilin Agricultural University, where they were maintained under standard conditions (humidity: 51–13%, temperature: 23 ± 3°C, light–dark cycle: 12/12). Following a period of adaptive feeding lasting 7 days, the mice were categorically assigned to one of four groups: (i) Control group (C, *n* = 28), which received standard feeding without additional interventions. (ii) Inflammatory control group (E, *n* = 28), where vaginal perfusion with normal saline occurred from gestational days 17.5 to 19.5, followed by uterine infusion with bovine pathogenic *E. coli* on the first and second days postpartum. *E. coli* was isolated from cows with endometritis, and the *E. coli*-induced endometritis mouse model was established following the protocol outlined in our previous studies (20, 21). Briefly, 25 μL of a mixed *E. coli* suspension (1 × 10^10^ CFU/mL) was inoculated into the uterus of anesthetized mice using a 19 mm soft needle (outer diameter 0.7 mm) to induce endometritis. (iii) *L. johnsonii* group (Lc, *n* = 34), subjected to vaginal perfusion with bacterial fluid from gestational days 17.5 to 19.5, and uterine infusion with pathogenic *E. coli* on the first and second days postpartum. Five hundred microliters of *johnsonii* (1 × 10^7^ CFU/mL) was used in this study. (iv) Recombinant *L. johnsonii* group (Lc-GM-CSF, *n* = 32), receiving the same treatment as the Lc group.

### 2.6 Histomorphological analysis

The uterine tissue specimens were isolated, fixed in 4% buffered formaldehyde, and subsequently subjected to standard paraffin embedding for a duration exceeding 48 h. Following this, the samples underwent staining utilizing conventional hematoxylin-eosin (H&E) techniques. Cross-sectional visualization and analysis of the uterine tissue were performed using a light microscope (Olympus BX41, Olympus Optical Co., Ltd., Tokyo, Japan) and evaluated with Image-Pro Plus 6.0 software (Media Cybernetics, Bethesda, United States).

### 2.7 Reverse transcription-quantitative polymerase chain reaction assay

Total RNA was extracted from uterine tissues using the Trizol reagent (Gibco BRL; Thermo Fisher Scientific) in accordance with the manufacturer’s instructions. Subsequently, the RNA obtained from each sample was reverse transcribed into complementary DNA (cDNA) utilizing the PrimeScript^™^ RT reagent Kit with gDNA Eraser (Takara Biotechnology Co., Ltd.). The reverse transcription reaction conditions were as follows: genomic DNA elimination at 42°C for 2 min, reverse transcription at 37°C for 15 min, and inactivation of the reverse transcriptase at 85°C for 5 s. The subsequent step involved performing Quantitative Real-Time PCR using a 7500HT fast real-time PCR system (ABI; Thermo Fisher Scientific, Inc.). The amplification conditions were as follows: pre-denaturation at 95°C for 30 s, followed by 40 cycles of PCR reaction with denaturation at 95°C for 5 s, annealing at 60°C for 30 s. The relative expression levels were determined utilizing the 2^−ΔΔCT^ Cq method. The primers employed for the reverse transcription-quantitative polymerase chain reaction (RT-qPCR) analysis were shown in Table 1.

**Table 1:** Primer information.

Gene name	Sequence (5′–3′)
*GM-CSF*	F: ATGTGGCTGCAGAACCTGCT
	R: TCACTTCTGGGCTGGTTCCC
*IL-6*	F: CCACTTCACAAGTCGGAGGCTTA
	R: CCAGTTTGGTAGCATCCATCATTTC
*IL-1β*	F: TCCAGGATGAGGACATGAGCAC
	R: GAACGTCACACACCAGCAGGTTA
*TNF-α*	F: TATGGCCCAGACCCTCACA
	R: GGAGTAGACAAGGTACAACCCATC
*β-actin*	F: TCAGGTCATCACTATCGGCAAT
	R: AAAGAAAGGGTGTAAAACGCA

### 2.8 The enzyme-linked immunosorbent assay

Mice uterine tissue weighing 1 g was excised, immersed in 9 mL of phosphate-buffered saline (pH 7.2–7.4), and subjected to triple washes. The resulting wash samples were then collected and centrifuged at 4,000 rpm for 20 min at 4°C. The supernatants were collected and preserved at −20°C until further analysis. Bovine blood samples were obtained and subsequently centrifuged at 2,000 rpm for 15 min at 4°C, followed by storage at −80°C for subsequent analysis. Cotton swabs containing bovine uterine mucus were added to 300 μL PBS, shake for 2 min, freeze and thaw repeatedly in liquid nitrogen three times, centrifuge at 4°C 12,000 rpm for 15 min, collected the supernatant for further analysis. Commercial enzyme-linked immunosorbent assay (ELISA) kits (BYabscience) were employed for the assessment of IL-6, IL-1β and TNF-α protein expression levels. Absorbance values were read at 450 nm. Determination of myeloperoxidase (MPO) content and NO content in mouse uterine tissue.

The level of myeloperoxidase was assessed using the myeloperoxidase assay kit (procured from Nanjing Jiancheng Biology Co., Ltd., Nanjing, China), with absorbance readings at 450 nm conducted using a spectrophotometer. Uterine tissue samples were collected and homogenized for subsequent analysis of NO concentration in the supernatant, employing the NO assay kit (Solarbio Life Science, Beijing, China).

### 2.9 Data analysis

Statistical analyses were conducted using the one-way ANOVA program in SPSS22, while GraphPad Prism 7 was employed for further statistical analyses. The data were presented as mean ± standard deviation (SD). For multiple comparisons, one-way ANOVA was performed, followed by Duncan’s *post hoc* test. The data are expressed as the mean ± SD. Statistical significance was set at ^*^p < 0.05.

## 3 Results

### 3.1 Construction of the recombinant *Lactobacillus johnsonii* expressing bovine GM-CSF

The gene encoding bovine GM-CSF (456 bp) was artificially synthesized and inserted into the vector PUC57. Following this, specific primer pairs (Table 1) were utilized to amplify the GM-CSF fragment from the PUC57-GM-CSF, which was then inserted into the expression vector pPG612, resulting in the generation of the recombinant plasmid pPG612-GM-CSF (Figures 1A,B). Validation of this process was achieved through restriction enzyme digestion and sequencing (Figure 1C), confirming the successful construction of recombinant plasmid pPG-GM-CSF.

Subsequently, these recombinant plasmids were inserted into *L. johnsonii* via electroporation, constructed the recombinant *L. johnsonii* expressing bovine GM-CSF (Lc-pPG-GM-CSF). As depicted in Figure 1D, the PCR results confirmed the successful transfer of the recombinant plasmid pPG612-GM-CSF into *L. johnsonii*, and sequencing result confirmed that the inserted sequence corresponds to bovine GM-CSF (456 bp). Moreover, the stability of the recombinant *L. johnsonii* pPG612-GM-CSF over 40 generations is demonstrated in lanes 1–5 of Figure 1E, providing evidence of its successful inheritance. Finally, Western blotting revealed a 15 kDa band corresponding to the GM-CSF protein in the cell lysates of Lc-pPG-GM-CSF, confirming the successful construction of the *L. johnsonii* expressing bovine GM-CSF (Figure 1F).

### 3.2 Recombinant *Lactobacillus johnsonii* expressing GM-CSF decreased incidence rate in mice model

**Figure 2:**
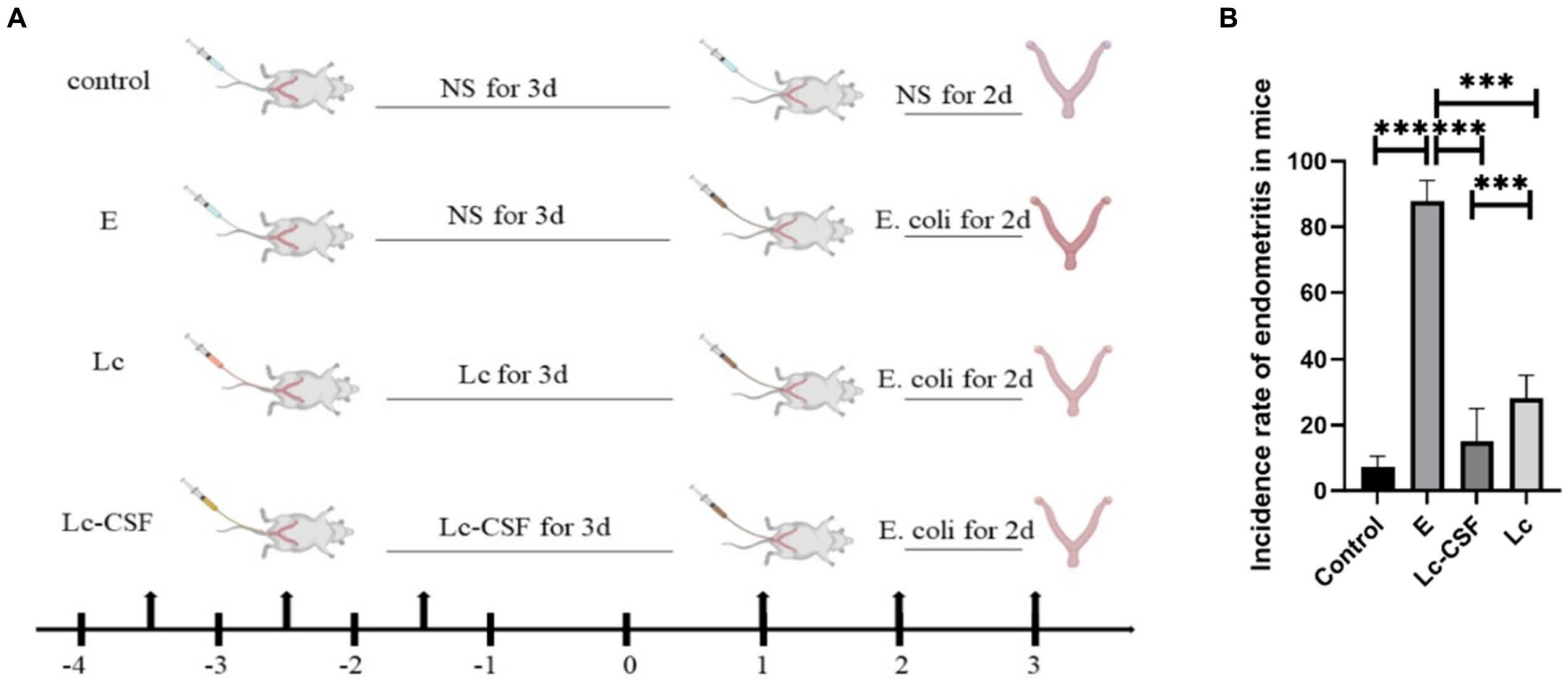
Recombinant *L. johnsonii* provides significant protection against *E. coli*-induced postpartum endometritis in mice model. **(A)** Immune and construction process of a mouse model of postpartum endometritis. **(B)** The incidence of postpartum endometritis in C, E, Lc, Lc-GM-CSF group. One-way ANOVA analysis was used for statistical analysis and all data are presented as mean ± standard deviation (SD). ns, *p* > 0.05, ^*^p < 0.05, ^**^p < 0.01, and ^***^p < 0.001.

We used the *E. coli* perfusion method to establish a mouse postpartum endometritis model, reported in our previous studies (20, 21). The immunological and constructive process of the postpartum endometritis mice model is shown in Figure 2A. Following two consecutive days of perfusion with *E. coli*, a statistical analysis was conducted to assess the incidence of postpartum endometritis in the respective mouse groups (Figure 2B). During the development and administration of the model, mice encompassed a control group (C, *n* = 28), an *E. coli* perfusion control group (E, *n* = 28), an *L. johnsonii*-immunization group (Lc, *n* = 34), and a recombinant *L. johnsonii*-immunization group (Lc-GM-CSF, *n* = 32). The results showed a substantial disparity, with the incidence rate in group E reaching 87.86 ± 6.23%. In contrast, the Lc-GM-CSF group exhibited a remarkable reduction in the incidence rate, dropping to 15.14 ± 9.9%. However, the incidence rate in the Lc group was 28.09% ± 7.02%, which is significantly higher than that of the Lc-GM-CSF group (*p* > 0.05). These findings underscore the significant protective efficacy of recombinant *L. johnsonii* expressing GM-CSF against *E. coli*-induced postpartum endometritis.

### 3.3 Recombinant *Lactobacillus johnsonii* expressing GM-CSF diminished uterine pathological damage and histopathological alterations in mice model

**Figure 3:**
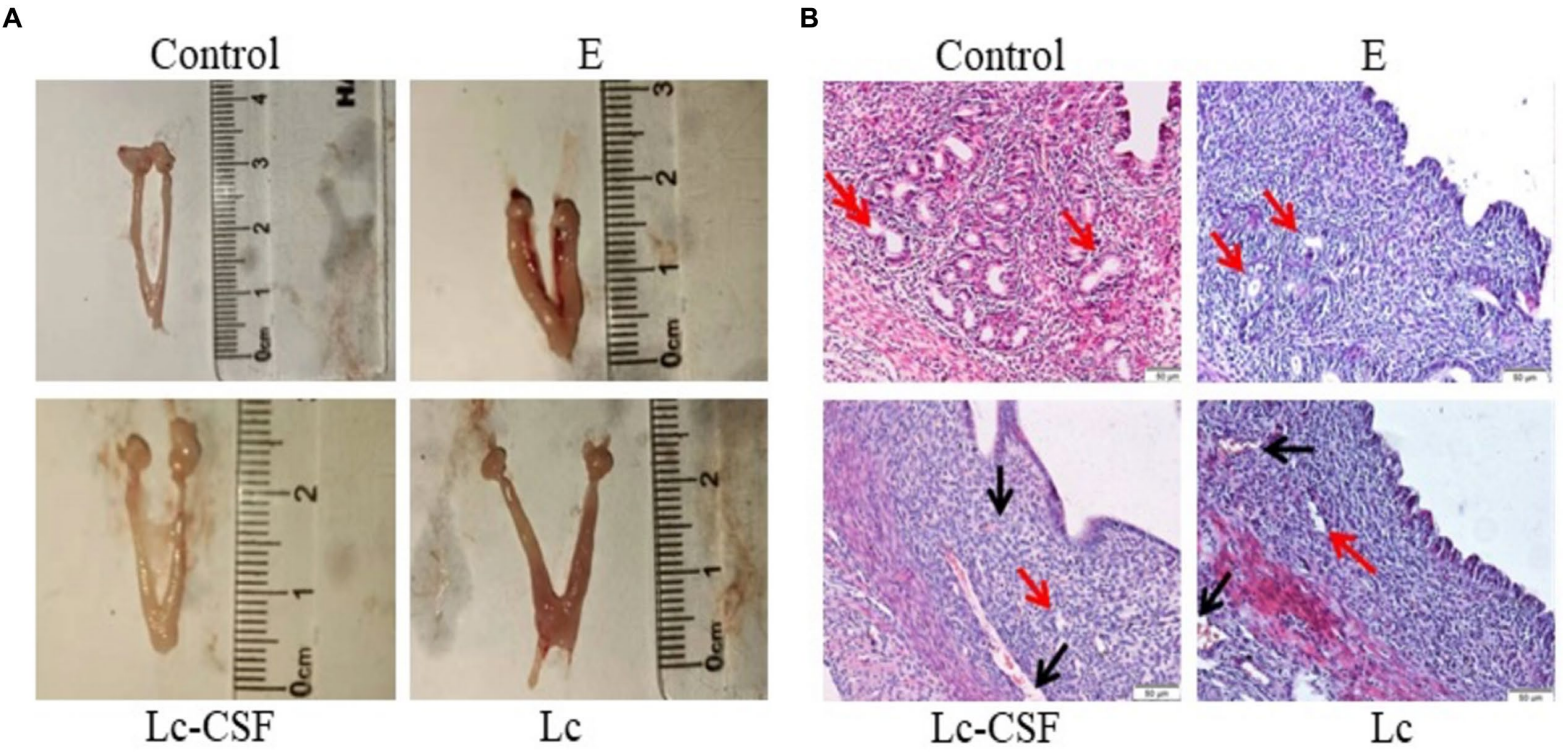
The effect of Lc-pPG-GM-CSF on the morphology of uterine tissue in mice with endometritis. **(A)** Morphological observation of mouse uterus after three days of delivery. **(B)** H&E staining was used to observe the uterus of mice three days after delivery (magnification: ×200). C group, Control group; E group, an inflammatory control group; Lc group, vaginal perfusion with *L. johnsonii* from gestational days 17.5 to 19.5, and uterine infusion with pathogenic *E. coli* on the first and second days postpartum; Lc-GM-CSF group, vaginal perfusion with recombinant *L. johnsonii* expressing GM-CSF from gestational days 17.5 to 19.5, and uterine infusion with pathogenic *E. coli* on the first and second days postpartum. The red arrow represents uterine glands, and the black arrow represents capillaries.

Three days postpartum, uterine specimens were obtained and their morphological characteristics are shown in Figure 3A. As illustrated, the uteri in the *E. coli* group exhibited engorged, edema, and hypertrophy, accompanied by a diminished gland. Conversely, uteri in the control group displayed a normal appearance. The uteri in the Lc group had a similar length to the control group, but showed signs of redness, swelling, and increased thickness compared to the control group. The uteri in the Lc-GM-CSF group shared a similar length to the control group, and notably, evinced a substantial mitigation of engorged and edema compared to the *E. coli* group, approaching a normative uterine state.

The uterine morphological alterations are depicted in Figure 3B. Within the control cohort, a state of normal tissue morphology prevailed, characterized by a distinct demarcation between the endometrium and the myometrium, the number and morphology of the gland was normal. Conversely, the *E. coli* group exhibited conspicuous endometrial hyperemia and interstitial edema, along with an indistinct boundary between the endometrium and the myometrium, a reduction in glandular abundance, and partial exfoliation of the uterine epithelium. In the Lc group, although amelioration of hyperemia was observed, vestiges of hyperemic regions persisted, accompanied by the presence of a limited number of glands. The Lc-GM-CSF group showed close-to-normal uterine morphology, devoid of any hyperemic regions and showing discernible glandular structures. Following *E. coli*-induced inflammation, mice subjected to recombinant *L. johnsonii* expressing GM-CSF displayed diminished uterine pathological damage and histopathological alterations.

### 3.4 Recombinant *Lactobacillus johnsonii* expressing GM-CSF decreased the production of pro-inflammatory factors in *Escherichia coli*-induced postpartum endometritis mice

**Figure 4:**
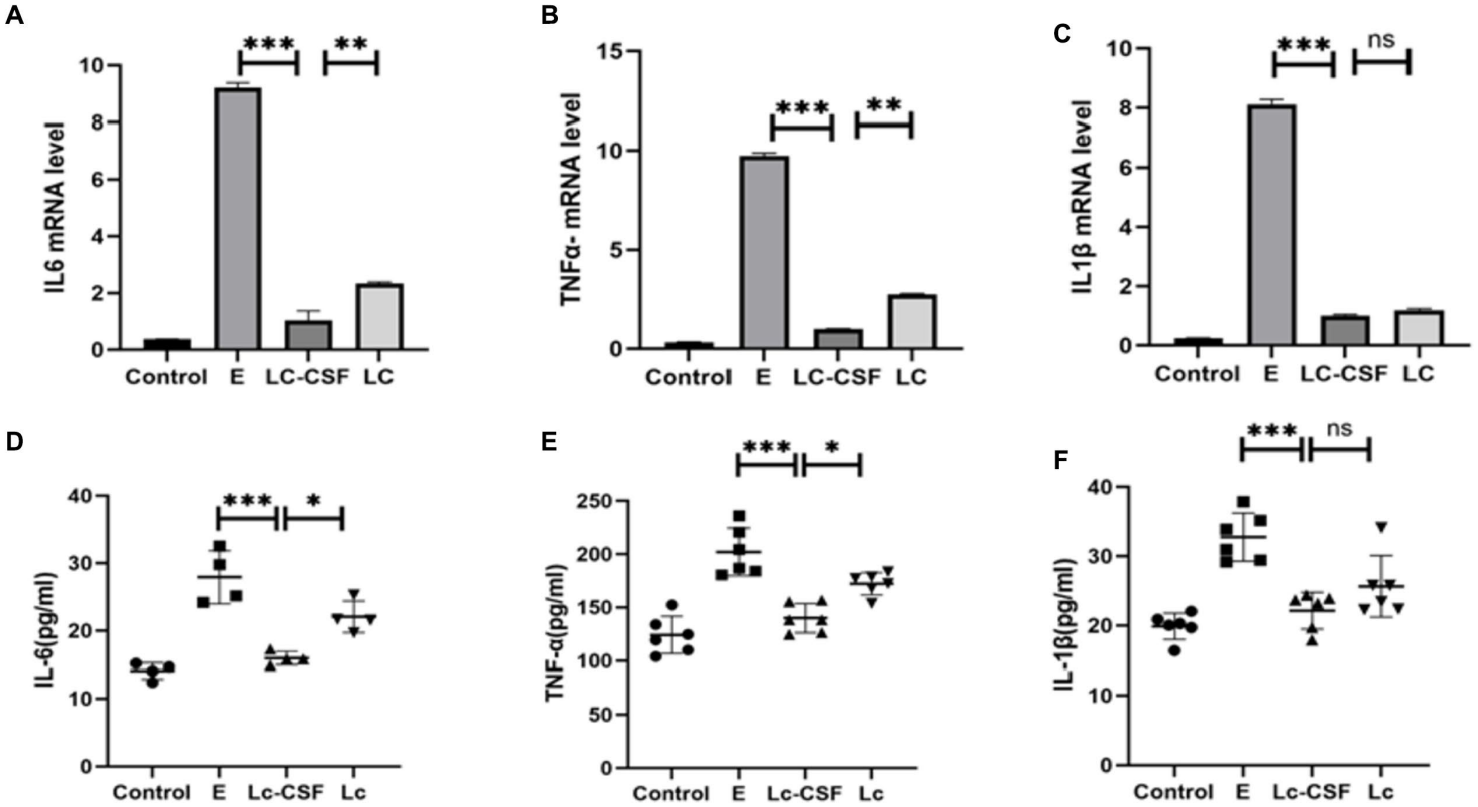
Effects of Lc-pPG-GM-CSF on the expression of pro-inflammatory factors at the protein and gene levels in mice with endometritis. Effects of Lc-pPG-GM-CSF on the expression of IL-6 **(A)**, TNF-α **(B)** and IL-1β **(C)** in the uterine tissue of mice with endometritis at the gene level. Effect of Lc-pPG-GM-CSF on the expression of IL-6 **(D)** TNF-α **(E)** and IL-1β **(F)** in the uterine tissue of mice with endometritis at the protein level. C group, Control group; E group, an inflammatory control group; Lc group, vaginal perfusion with *L. johnsonii* from gestational days 17.5 to 19.5, and uterine infusion with pathogenic *E. coli* on the first and second days postpartum; LcGM-CSF group, vaginal perfusion with recombinant *L. johnsonii* expressing GM-CSF from gestational days 17.5 to 19.5, and uterine infusion with pathogenic *E. coli* on the first and second days postpartum. One-way ANOVA analysis was used for statistical analysis and all data are presented as mean ± standard deviation (SD). ns, *p* > 0.05, ^*^p < 0.05, ^**^p < 0.01, and ^***^p < 0.001.

To further investigate the impact of recombinant *L. johnsonii* expressing GM-CSF on pro-inflammatory factors, we conducted an analysis on IL-6, IL-1β, and TNF-α at both the mRNA (q-PCR) and protein (ELISA, sensitivity: 0.1 pg/mL) levels of the mice uterine tissue. The mRNA and protein expressions of IL-6, IL-1β, and TNF-α were markedly elevated in the *E. coli* group (Figure 4). In contrast, the Lc group exhibited a substantial reduction in the expression of inflammation-associated mRNA and proteins when compared to the *E. coli* group (*p* < 0.001). Moreover, the Lc-GM-CSF group demonstrated a significant decrease in mRNA expression of IL-6 and TNF-α, as well as protein expressions of IL-6, IL-1β, and TNF-α, compared to the Lc group (*p* < 0.005). These findings suggested the potent anti-inflammatory effects of *Lactobacillus* interventions in mitigating the inflammatory response.

### 3.5 Recombinant *Lactobacillus johnsonii* expressing GM-CSF reduced MPO activity and NO concentration

**Figure 5:**
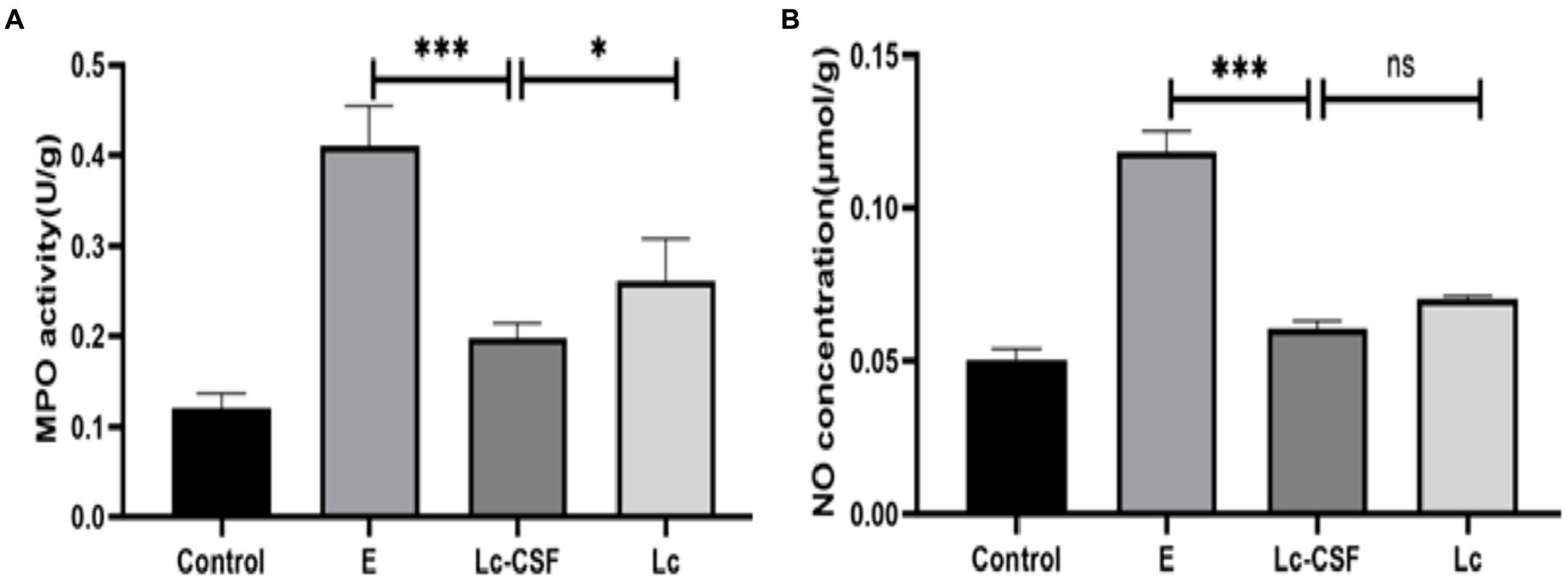
Effects of Lc-pPG-GM-CSF on the inflammatory response in mice with endometritis. Effect of Lc-pPG-GM-CSF on the MPO activity **(A)** and NO concentration **(B)** in the uterine tissue of mice with endometritis. C group, Control group; E group, an inflammatory control group; Lc group, vaginal perfusion with *L. johnsonii* from gestational days 17.5 to 19.5, and uterine infusion with pathogenic *E. coli* on the first and second days postpartum; Lc-GM-CSF group, vaginal perfusion with recombinant *L. johnsonii* expressing GM-CSF from gestational days 17.5 to 19.5, and uterine infusion with pathogenic *E. coli* on the first and second days postpartum. One-way ANOVA analysis was used for statistical analysis and all data are presented as mean ± standard deviation (SD). ns, *p* > 0.05, ^*^p < 0.05, ^**^p < 0.01, and ^***^p < 0.001.

The heightened myeloperoxidase (MPO) activity and augmented nitric oxide (NO) concentration serve as pivotal indicators of inflammatory pathologies, with alterations in their levels reflecting the extent of oxidative stress. Following uterine exposure to pathogenic *E. coli*, a notable increase in both MPO activity (Figure 5A) and NO concentration (Figure 5B) (*p* < 0.05) was observed compared to the Control group. Conversely, mice vaginal perfusion with Lc-GM-CSF demonstrated reduced MPO activity (Figure 5A) and NO concentration (Figure 5B) relative to the *E. coli* group and Lc group (*p* < 0.05). These observations indicate the potential of Lc-GM-CSF in the mitigating impact of uterine *E. coli*-induced inflammation by regulating MPO activity and NO concentration.

### 3.6 Recombinant *Lactobacillus johnsonii* expressing GM-CSF decreased the level of cytokines involved in inflammation of bovine endometrium

**Figure 6:**
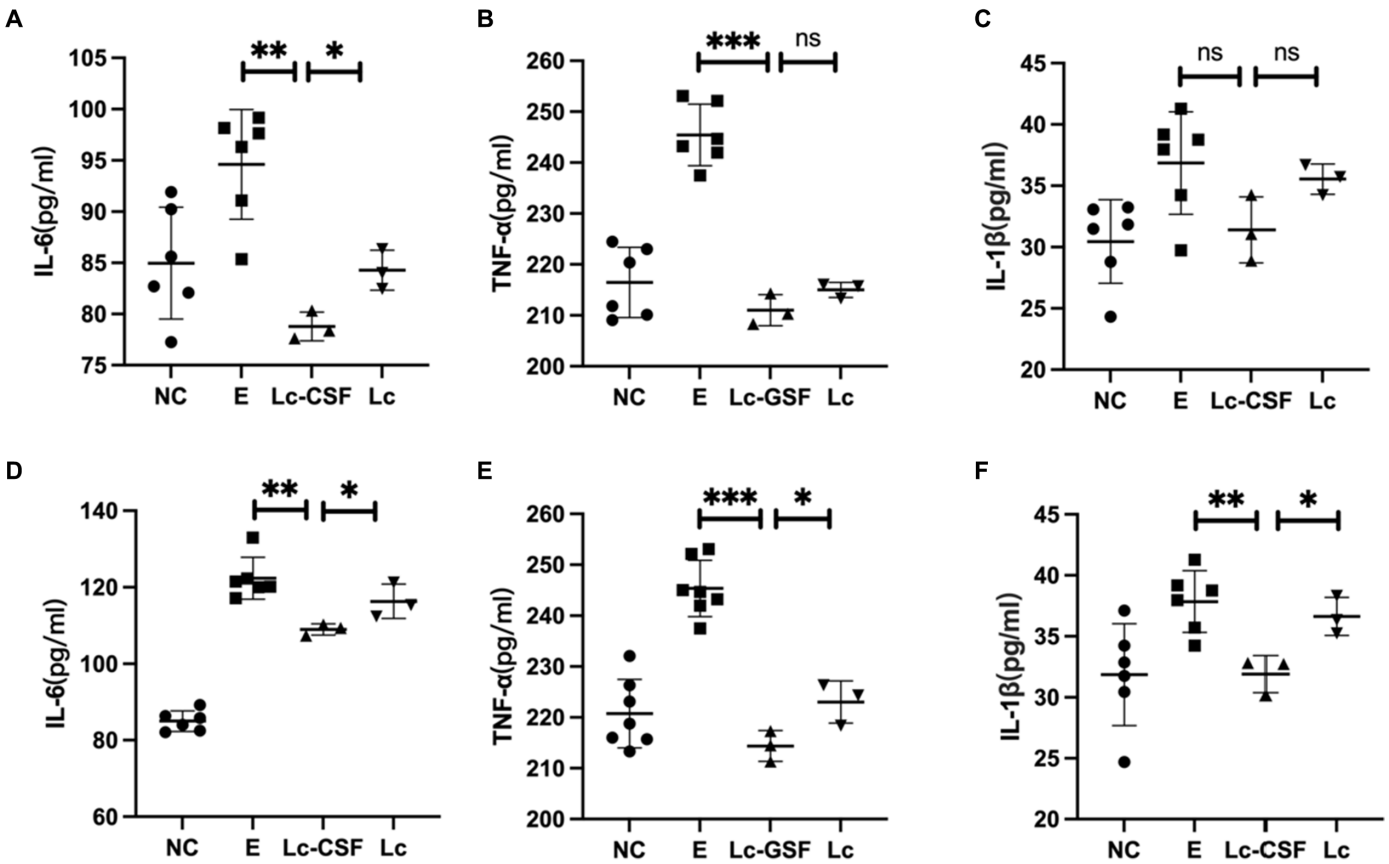
Effects of Lc-pPG-GM-CSF on the expression of pro-inflammatory factors at the protein and gene levels in cows with endometritis. Effect of Lc-pPG-GM-CSF on the expression of IL-6 **(A)**, TNF-α **(B)**, and IL-1β **(C)** in the uterine mucus of cows. Effect of Lc-pPG-GM-CSF on the expression of IL-6 **(D)**, TNF-α **(E)**, and IL-1β **(F)** in the serum of cows with endometritis. C group, Control group; E group, an inflammatory control group; Lc group, vaginal perfusion with *L. johnsonii* from gestational days 17.5 to 19.5, and uterine infusion with pathogenic *E. coli* on the first and second days postpartum; Lc-GM-CSF group, vaginal perfusion with recombinant *L. johnsonii* expressing GM-CSF from gestational days 17.5 to 19.5, and uterine infusion with pathogenic *E. coli* on the first and second days postpartum. One-way ANOVA analysis was used for statistical analysis and all data are presented as mean ± standard deviation (SD). ns, *p* > 0.05, ^*^p < 0.05, ^**^p < 0.01, and ^***^p < 0.001.

To further evaluate the therapeutic effect of recombinant *L. johnsonii* on bovine endometritis, we conducted further investigation of pro-inflammatory factors in protein (ELISA) levels on bovine serum and uterine mucus from control and the treatment group. The protein expression levels of pro-inflammatory cytokines (IL-1, IL-6, and TNF-α) after treatment for clinical endometritis is presented in Figure 6. The highest serum and mucus concentrations of pro-inflammatory cytokines were observed in the E group (bovine endometritis group). While all studied pro-inflammatory cytokines showed a decrease in the treatment group (Lc and Lc-GM-CSF). Moreover, the LC-GM-CSF group (recombinant *L. johnsonii* treatment group) had the lowest concentrations (*p* < 0.005), similar to the normal control. These findings indicate that recombinant *L. johnsonii* expressing GM-CSF exert a favorable influence by decreasing the level of cytokines involved in endometritis inflammation.

## 4 Discussion

Endometritis has always been an important disease causing reproductive disorders in dairy cows (22). In the research related to the treatment of bovine endometritis, the application of antibiotic treatment is relatively extensive, but in the long run, the combined use of antibiotics will often accelerate the drug resistance of pathogens and lead to antibiotic residues in milk, bringing huge food safety risks to the health of consumers (23). Therefore, it is an urgent problem for clinical treatment to avoid the side effects of antibiotics in treating bovine endometritis. In this study, we successfully constructed a recombinant *Lactobacillus johnsonii* strain expressing bovine GM-CSF, which was found to significantly reduce inflammation levels induced by *E. coli* infection postpartum endometritis mice model and demonstrated a significant therapeutic effect on cow endometritis.

Lactic acid bacteria or their extracts and metabolites trigger the innate immune response and activate NF-κB by stimulating TNF-α signaling in epithelial cells (24). Meanwhile, GM-CSF has a potential stimulating function on the expansion and maturation of monocyte-macrophages, dendritic cells, and granulocytes derived from hematopoietic progenitor cells. Thus, we combined lactic acid bacteria and GM-CSF. A recombinant plasmid pPG612-GM-CSF was successfully constructed and introduced into *L. johnsonii*. A 15 kDa band corresponding to the GM-CSF protein was detected in the cell lysate of recombinant Lc-pPG-GM-CSF, indicating that the bovine GM-CSF was indeed expressed in the recombinant lactic acid bacteria. In addition, other studies have shown that GM-CSF can increase the production of antimicrobial agents, promote phagocytosis, and improve immune function (25–27). These results further suggest the potential protective effect of recombinant *L. johnsonii* expressing bovine GM-CSF against inflammation.

The immune system plays an important role in maintaining health and is crucial in resisting infections and defending tissue integrity (28, 29). In recent years, the immunomodulatory effects of probiotics as a novel therapeutic tool have been extensively studied (30). Lactic acid bacteria, a common probiotic, has been widely used in clinical settings and demonstrates significant advantages in immune regulation (31). Studies have shown that lactic acid bacteria can affect host immune responses through the production of metabolites such as lactic acid and bacteriocins (32). In addition, the presence of recombinant lactic acid bacteria can inhibit the expression levels of inflammation-related genes and proteins in uterine tissues, regulating immune function (19). We found that recombinant *L. johnsonii* (Lc-GM-CSF) can effectively prevent the occurrence of uterine infection in mouse models, reduce cytokine expression levels, specifically, lower the cytokines expression levels of clinical cow endometritis significantly, demonstrating a significant therapeutic effect on the disease. Cytokines (IL-1β, IL-6 and TNF-α) are key regulatory factors in the inflammatory response, and their abnormal expression is closely related to the development and progression of inflammation. Our finding indicated that recombinant lactic acid bacteria may play a protective role in endometritis by regulating the expression of these inflammation-related genes.

GM-CSF has a crucial protective role in inflammation-induced postpartum endometritis. When stimulated by LPS, all the extra-cervical cells, intra-cervical cells, and mesenchymal stem cells in amniotic fluid produce GM-CSF (33). Upregulation of GM-CSF in the cervix and uterus was also observed in a mouse model of postpartum endometritis. Importantly, the use of exogenous GM-CSF drugs reduces the incidence of inflammation-induced postpartum endometritis (34). Recombinant GM-CSF *lactobacillus*, with the dual immunomodulatory effects of *lactobacillus* and GM-CSF, can reduce the inflammatory response and release of inflammatory mediators in postpartum endometritis by inhibiting the activation and aggregation of neutrophils (35). For example, recombinant GM-CSF *lactobacillus* may reduce the production of MPO by regulating the activation state of neutrophils (36). In addition, recombinant GM-CSF *lactobacillus* may inhibit the inflammatory response by regulating the release of NO from neutrophils. NO released by neutrophils can serve as an important inflammatory mediator, and its inhibition may help alleviate the severity of postpartum endometritis (37). Consistent with the above researches, we found that in the recombinant GM-CSF *lactobacillus* group, both MPO activity and NO concentration in uterine tissue were significantly decreased. This suggests that recombinant *lactobacillus* may enhance the function of neutrophils, and reduce the inflammatory response, and oxidative stress.

**Figure 7:**
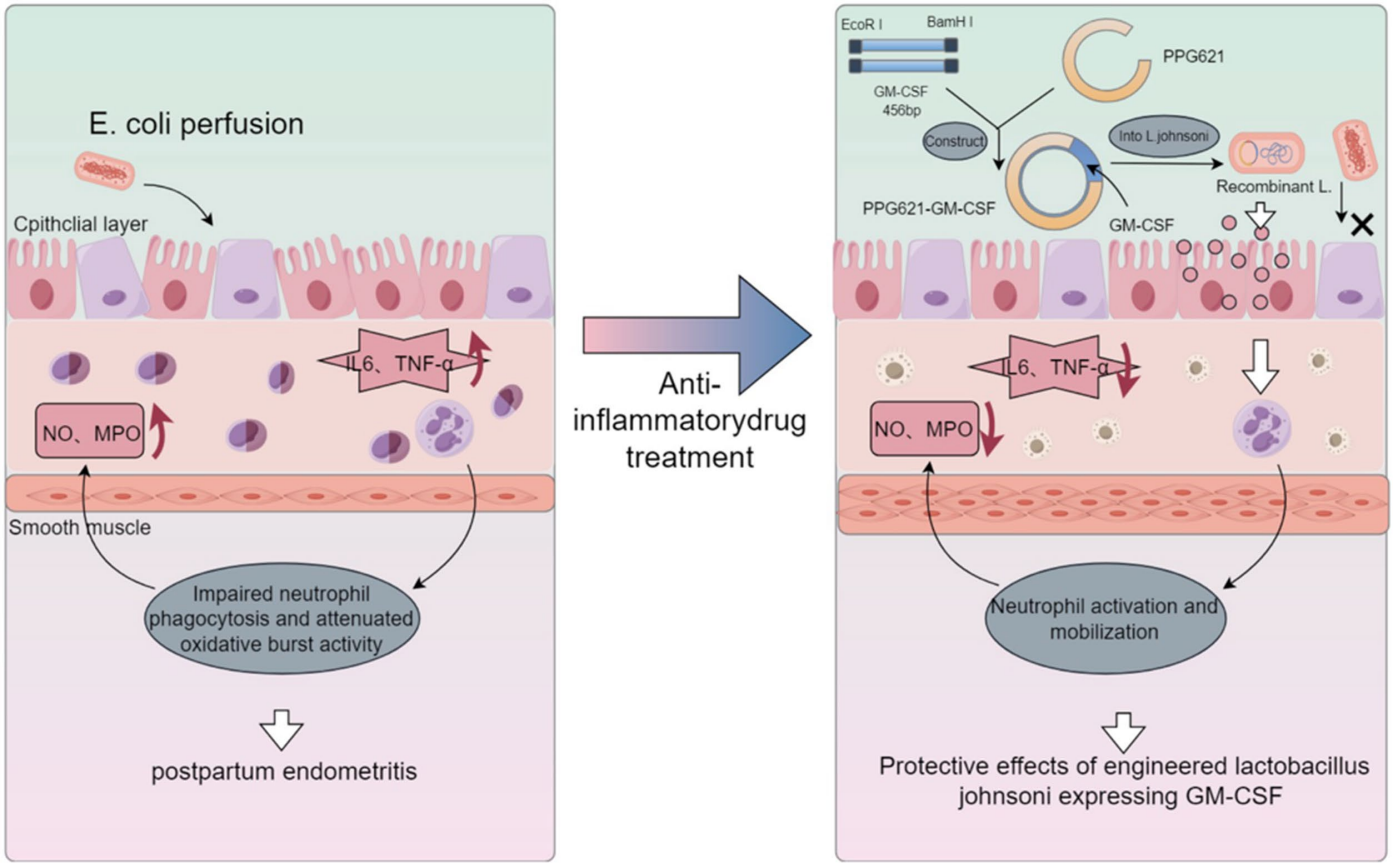
Protective effects of engineered *Lactobacillus johnsonii* expressing bovine GM-CSF on bovine postpartum endometritis. We successfully constructed a recombinant *Lactobacillus johnsonii* strain that expresses bovine granulocyte-macrophage colony-stimulating factor (GM-CSF), which significantly reduced the incidence and inflammation levels of endometritis in a mouse model, as well as exerted beneficial effects on bovine endometritis, presumably through the stimulation of neutrophil function. These findings suggest that the recombinant *Lactobacillus johnsonii* strain expressing bovine GM-CSF has potential clinical application value for the prevention and treatment of postpartum uterine inflammation.

In conclusion, this study elucidates the protective effect of recombinant *L. johnsonii* expressing bovine GM-CSF against postpartum endometritis (Figure 7). The recombinant plasmid was successfully constructed and introduced into *L. johnsonii*, resulting in the expression of bovine GM-CSF protein. We found that recombinant *L. johnsonii* inhibited the expression of inflammation-related genes and proteins in the uterine tissues of the mouse model, suggesting a role in regulating immune function. Furthermore, recombinant *L. johnsonii* demonstrated a significant therapeutic effect on cow endometritis. However, further studies are needed to advance the potential clinical application of an engineered strain that carries GM-CSF for bovine disease treatment. Nevertheless, our findings provide a new strategy for the prevention and treatment of bovine endometritis.

## Data availability statement

The original contributions presented in the study are included in the article/supplementary material, further inquiries can be directed to the corresponding authors.

## Ethics statement

The animal study was approved by Animal Management Committee, Jilin Agricultural University. The study was conducted in accordance with the local legislation and institutional requirements.

## Author contributions

JG: Formal analysis, Methodology, Writing – original draft. XC: Formal analysis, Methodology, Writing – original draft. ZL: Project administration, Resources, Writing – review & editing. CW: Project administration, Resources, Writing – review & editing. CZ: Investigation, Methodology, Writing – review & editing. SW: Methodology, Writing – review & editing. ZF: Methodology, Writing – review & editing. JZ: Formal analysis, Writing – review & editing. JW: Investigation, Writing – review & editing. YF: Formal analysis, Writing – review & editing. HL: Investigation, Writing – review & editing. HD: Investigation, Writing – review & editing. XM: Funding acquisition, Writing – review & editing. WL: Funding acquisition, Writing – review & editing.
